# Tricks of the Types

**Published:** 1861-10

**Authors:** 


### TRICKS OF THE TYPES.

WE don't like to complain of the types, especially as we do not write as legibly as we once did. But, reader, we didn't make a "conical," but a *crucial* incision in that tumor we told you about, which closed the outlet of the parotid duct. And now, allow us to tell you that no further treatment proved necessary in that case. The recovery seems to be perfect.

And we didn't intend to say, in the September number, that the "invention" of pivot teeth is mechanical, but rather regarded the *insertion* of them as somewhat so. Being rather isolated, we don't always see our "proof," and will, therefore, not fret.

